# Origin of light instability in amorphous IGZO thin-film transistors and its suppression

**DOI:** 10.1038/s41598-021-94078-8

**Published:** 2021-07-16

**Authors:** Mallory Mativenga, Farjana Haque, Mohammad Masum Billah, Jae Gwang Um

**Affiliations:** 1 Department of Information Display, Kyung Hee University, Seoul, 02447 South Korea; 2 LG Electronics, Seoul, 17709 South Korea

**Keywords:** Engineering, Electrical and electronic engineering

## Abstract

Radiating amorphous In–Ga–Zn–O (a-IGZO) thin-film transistors (TFTs) with deep ultraviolet light (λ = 175 nm) is found to induce rigid negative threshold-voltage shift, as well as a subthreshold hump and an increase in subthreshold-voltage slope. These changes are attributed to the photo creation and ionization of oxygen vacancy states (V_O_), which are confined mainly to the top surface of the a-IGZO film (backchannel). Photoionization of these states generates free electrons and the transition from the neutral to the ionized V_O_ is accompanied by lattice relaxation, which raises the energy of the ionized V_O_. This and the possibility of atomic exchange with weakly bonded hydrogen leads to metastability of the ionized V_O_, consistent with the rigid threshold-voltage shift and increase in subthreshold-voltage slope. The hump is thus a manifestation of the highly conductive backchannel and its formation can be suppressed by reduction of the a-IGZO film thickness or application of a back bias after radiation. These results support photo creation and ionization of V_O_ as the main cause of light instability in a-IGZO TFTs and provide some insights on how to minimize the effect.

## Introduction

Light exposure has always been problematic for all thin-film transistors (TFTs)^1–3^ and amorphous-indium-gallium-zinc-oxide (a-IGZO) TFTs^4, 5^ are no exception. When combined with negative bias, near or above bandgap radiation of a-IGZO TFTs induces large negative threshold-voltage shift (ΔV_TH_), and numerous studies have been done to investigate the origin of this negative bias under illumination stress (NBIS) instability^6–15^. To date, there is no mechanism that has been universally agreed-upon and several mechanisms have been suggested to explain the negative ΔV_TH_, including trapping of photogenerated holes^8^ and/or creation of oxygen vacancy (V_O_)-related defects^10–15^.

Identifying the origin of the NBIS instability and reducing its effect is of paramount importance, given that the largest application for a-IGZO TFTs are displays, and the TFTs operate in the presence of light inside the displays. Although several methods to reduce defects in the a-IGZO films such as film thickness reduction^9^, treatments, or annealing in highly oxidative environments^12, 16–20^ have been proposed to reduce the effect of NBIS, light shields have been the most widely used^21^. However, alternatives to light shields are still required in applications forbidding the use of opaque metal layers, such as transparent displays used in Heads Up Displays (HUDs) or smart glasses. As negative bias applied in the absence of light is known to cause negligible changes in the performance of the a-IGZO TFTs^6, 8^, light is thus the major source of the NBIS instability.

**Figure 1:**
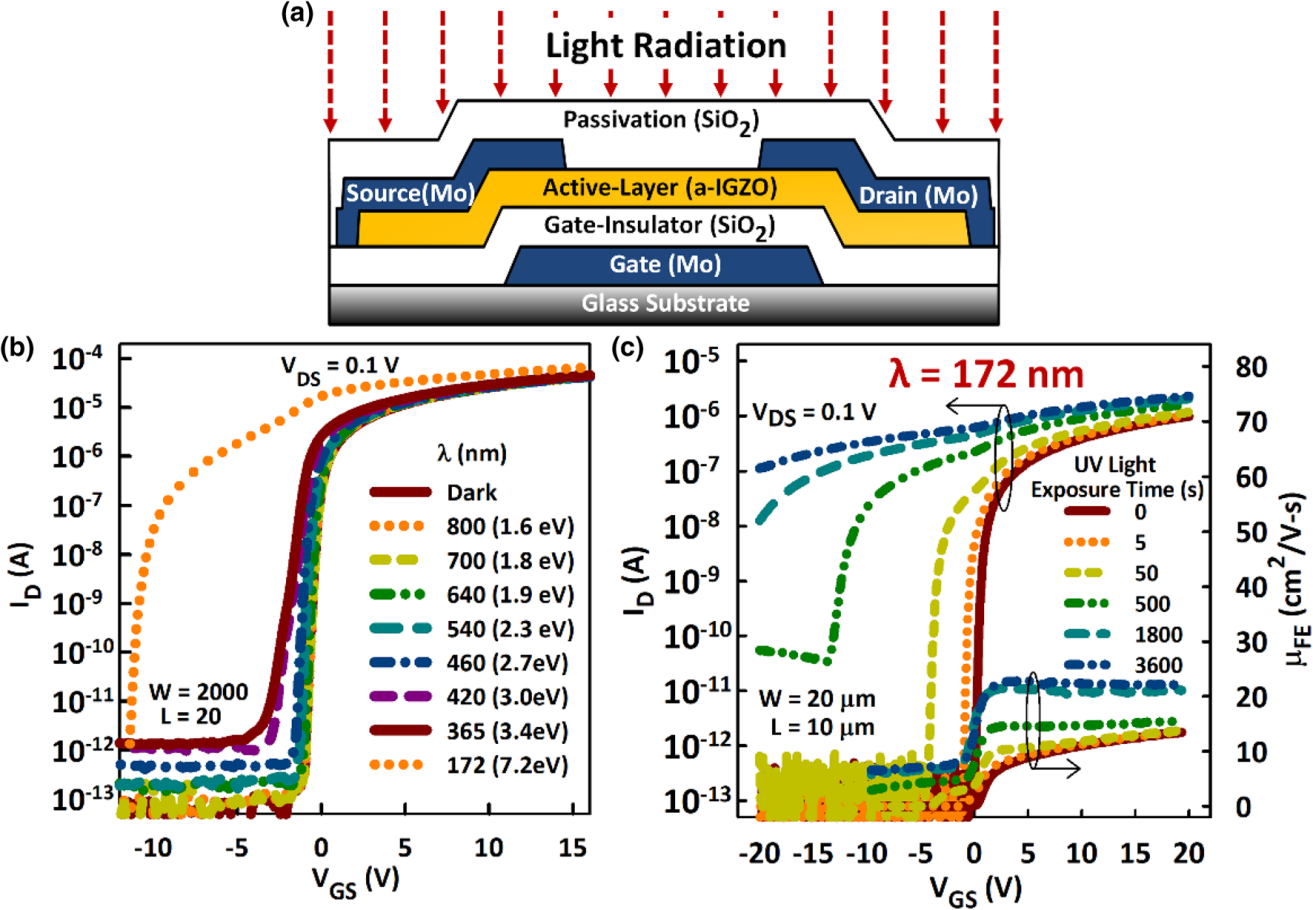
Effect of light radiation on the performance of a-IGZO TFTs. (**a**) Schematic cross-section of a-IGZO TFTs. (**b)** Transfer characteristics in dark and after radiation with monochromatic light of varying wavelength (λ) for 500 s. (**c**) Transfer characteristics and field-effect mobility (μ_FE_) as a function of exposure time to deep UV light (λ = 172 nm and intensity of 400 mW/cm^2^). Deep UV light radiation induces a large negative threshold voltage shift that is accompanied by a subthreshold hump and an increase in subthreshold voltage swing, as well as an increase in μ_FE._

In this study, we comprehensively investigate the effects of light radiation on a-IGZO TFTs in the absence of any electrical bias. We expose the a-IGZO TFTs (Fig. 1a) to monochromatic light of variable wavelength (800 to 172 nm). We show that photon energies less than 3.0 eV induce small changes in the performance of the a-IGZO TFTs, whereas deep UV light (7.2 eV) induces a large and rigid negative threshold-voltage shift (ΔV_TH_ > 20 V), as well as a subthreshold hump and an increase in the subthreshold-voltage slope (SS). Through Time-of-Flight Secondary Ion Mass Spectrometry (ToF–SIMS) and X-ray photoelectron spectroscopy (XPS), we also show that V_O_ states located at the top surface of the a-IGZO film are responsible for these changes. Radiated films contain a larger concentration of the V_O_ states than pristine films, indicating photo creation of the V_O_ states in addition to their photoionization.

Using TCAD (Technology Computer-Aided Design) simulation, we verify that the hump is a manifestation of the highly conductive backchannel and through the use of double gate TFTs with transparent top gates, we show that application of a back bias after light radiation suppresses the instability. Additionally, we show that the reduction of the a-IGZO film thickness in single gate TFTs can also suppress the light instability. These suppression methods are especially important for applications such as transparent displays where the use of light shields is not permissible. These results support photo creation and ionization of V_O_ as the main cause of light instability in a-IGZO TFTs and provide some insights on how to minimize the effect.

## Results and discussion

Effect of light radiation. Figure 1b shows the effects of monochromatic light on the performance of the a-IGZO TFTs. The wavelength of the light (λ) is varied from 800 to 172 nm, which corresponds to photon energy of 1.6 to 7.2 in electron volts (eV), and the transfer characteristics are measured after light irradiation for 500 s. Although small, the negative ΔV_TH_ after irradiation with photon energies less than 3.0 eV suggests photoexcitation from sub-gap states^22^. A slight jump in the ΔV_TH_ is apparent for photon energies in the range of 3.0–3.4 eV and indicates the onset of band-to-band excitation. The photon energy of the deep UV light (~ 7.2 eV) is large enough to induce substantial negative ΔV_TH_ (Fig. 1b). For a clear understanding of the effects of light in the a-IGZO TFTs, we therefore use the deep UV light (λ = 172 nm) in all investigations that follow. The negative ΔV_TH_ induced by deep UV light is accompanied by an increase in SS and the formation of a ‘hump’ in the transfer characteristics, which increases with exposure time (Fig. 1c). Also, quite noticeable is that the µ_FE_ doubles after radiating the TFTs with deep UV for 3,600 s, consistent with a photo-induced increase in free carrier concentration.

**Figure 2:**
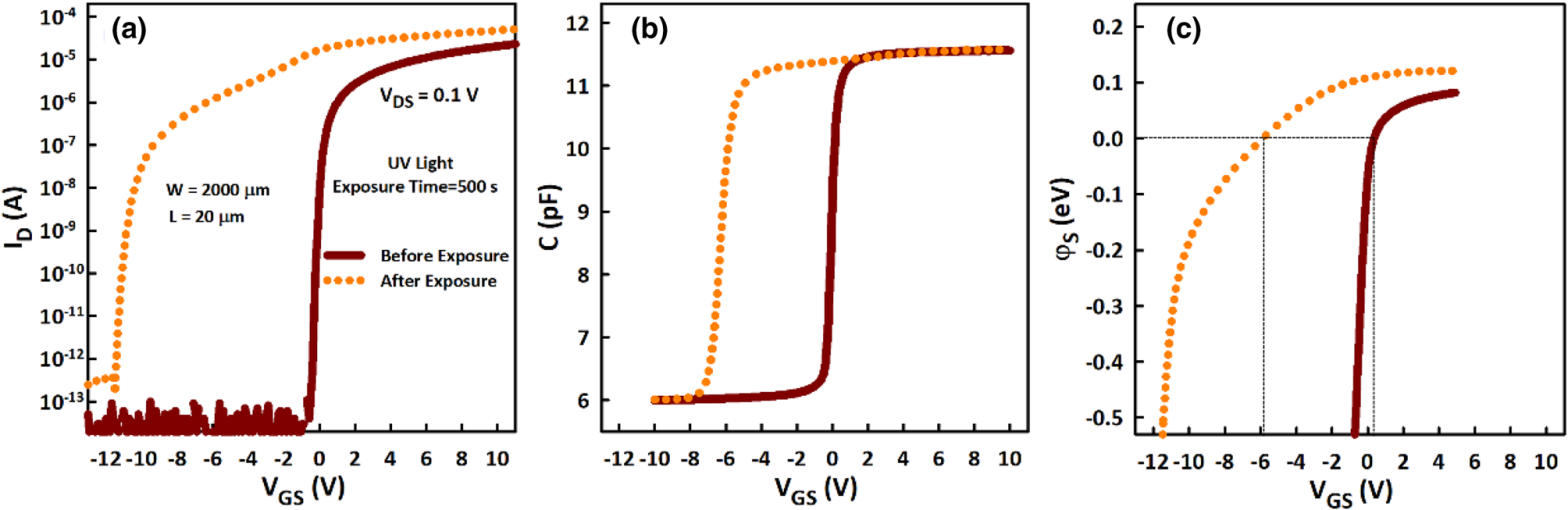
TFT characteristics used to determine the flat band parameters before and after deep UV light (λ = 172 nm) radiation. (**a)** Transfer (I_D_^–^V_GS_) characteristics. (**b**) Capacitance–voltage (C^–^V_GS_) characteristics. (**c**) Extracted surface potential (ψ_S_) as a function of V_GS_. The flat band voltage (V_FB_), which is taken as the V_GS_ corresponding to ψ_S_ = 0 eV, decreases from 0.36 to − 5.89 V after deep UV radiation.

Flat band parameters extracted from the combined analysis of the I_D_-V_GS_ (Fig. 2a) and C-V (Fig. 2b) characteristics are listed in Table 1. Detailed extraction methods can be found in Ref. 17. From the plot of surface potential (Ψ_S_) vs. V_GS_ (Fig. 2c), the flat band voltage (V_FB_), taken as the V_GS_ corresponding to Ψ_S_ = 0 V, decreases from 0.36 to − 5.89 V after light radiation. At the same time, the Fermi level (E_F_) moves closer to the conduction band (E_C_) by approximately 0.023 eV and flat band carrier concentration (n_FB_) increases from 1.4 × 10^16^ to 3.4 × 10^16^ cm^−3^. Interface trap density (N_int_, the algebraic sum of negative and positive interface charge density) also increases from − 7.7 × 10^10^ to 3.7 × 10^11^ cm^−2^ eV^−1^, while gap trap density per unit energy (dN_gap_/dE, the sum of interface and bulk density of states) increases from 1.3 × 10^12^ to 3.6 × 10^12^ cm^−2^ eV^−1^. Note that a negative value of N_int_ indicates that negative charge exceed positive charge by that amount and vice-versa, given that N_int_ is the algebraic sum of positive and negative interface charge density.

**Table 1:** Flat and parameters.

	E_C_–E_F_ (eV)	V_FB_ (V)	N_it_ cm^−2^ eV^−1^	n_FB_ (cm^−3^)	dN_gap_/dE (cm^−2^ eV^−1^)
Pristine	0.152	0.36	− 7.7 × 10^10^	1.4 × 10^16^	1.3 × 10^12^
After UV	0.129	− 5.89	3.7 × 10^11^	3.4 × 10^16^	3.6 × 10^12^

These photoinduced changes in µ_FE_, SS, n_FB_, V_FB_, E_F_–E_C_, N_it_, and dN_gap_/dE are all consistent with accumulation/trapping of net positive charge at the gate-insulator/a-IGZO interface and creation of defects in the a-IGZO layer. The increase in SS indicates formation of the defects and given that µ_FE_ increased and E_F_ moved closer to E_C_, some of these defects must be in the bulk of the a-IGZO and donor-like. The defects must also be positively charged (ionized), as the value of N_it_ changed from a negative to a positive number, making the increase in n_FB_ a sum of band-to-band and sub-gap photoexcitation.

### Recovery after UV light radiation

**Figure 3:**
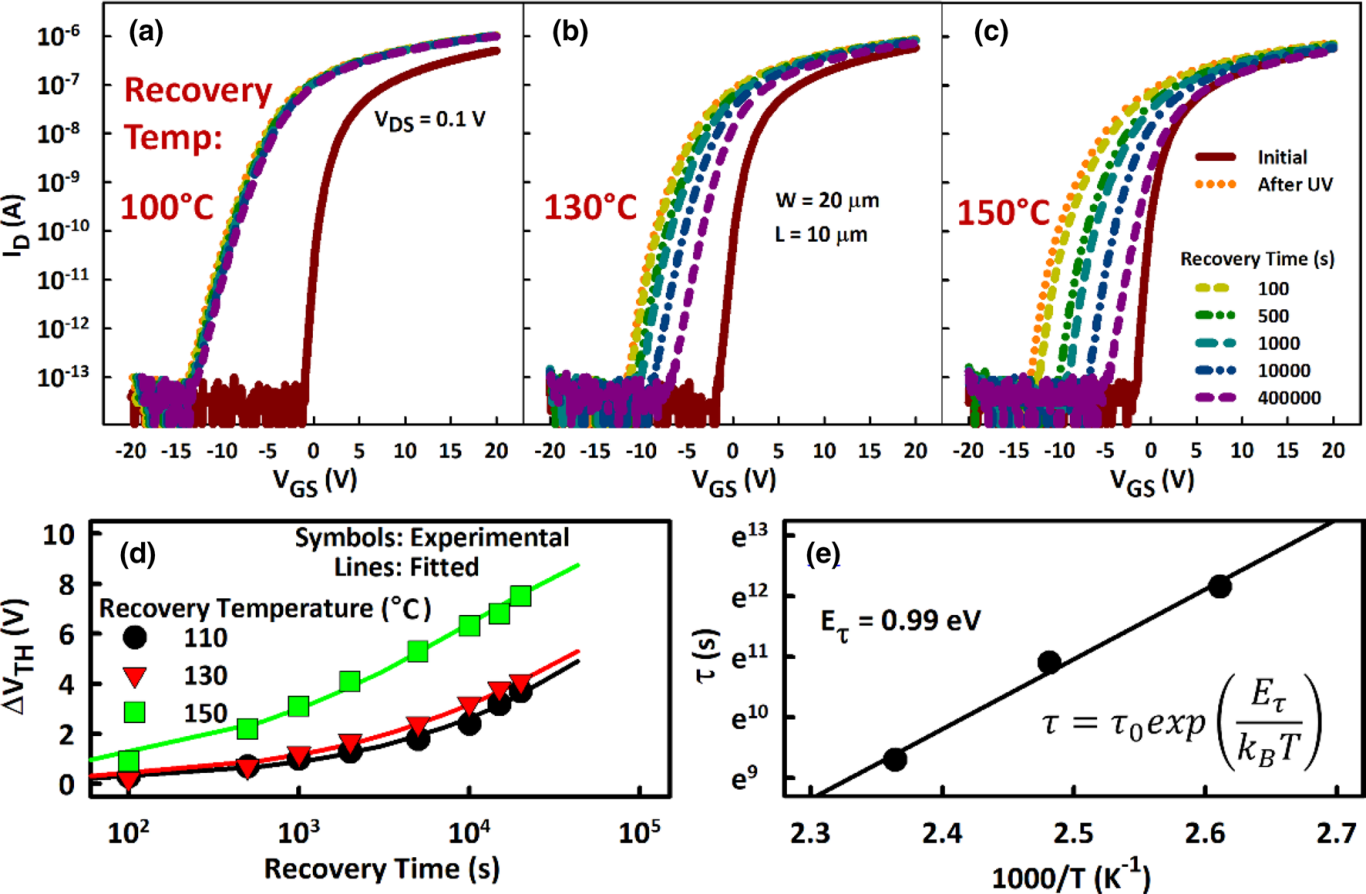
Recovery of transfer characteristics of a-IGZO TFTs at (**a**) 100 °C, (**b**) 130 °C, and (**c**) 150 °C after deep UV light (λ = 172 nm) radiation for 500 s. Recovery only occurs at temperature > 100 °C. (**d**) Threshold voltage shift (ΔV_TH_) as a function of recovery time from the deep UV light radiation stress. (**e**) Plot of the average time constant (τ) as an inverse function of temperature (Arrhenius plot). The activation energy for the recovery process (E_τ_ = 0.99 eV) is obtained from the slope of the straight line.

The photoinduced negative ΔV_TH_ is quite repeatable over many samples with varying channel dimensions. In particular, the ΔV_TH_ is independent of L (Supplementary Fig. S1) and rigid such that recovery in the dark is almost negligible at temperatures ≤ 100 °C (Fig. 3). At 150 °C, the transfer characteristics recover slowly and fail to return to the initial state even if the TFTs stay in the dark for a few days (Fig. 3c). Complete recovery is only achievable after annealing at 250 °C in vacuum for two or more hours. Therefore, the ionized donor-like defects generated by light radiation must be metastable states^23, 24^. However, the exceedingly small but rapid initial recovery (within 100 s) at low temperature could be due to delayed electron–hole recombination via non-metastable gap states.

As shown in the Fig. 3d, the time dependent ΔV_TH_ during the recovery period is well described by the stretched exponential equation given by

1 $$\left| {\Delta V_{TH} \left( t \right)} \right| = V_{O} \left\{ {1 - \exp \left[ { - \left( {\frac{t}{\tau }} \right)^{\beta } } \right]} \right\},$$

where V_0_ is the ΔV_TH_ at infinite time. The equation describes the superposition of processes with a spread of time constants (quantified by the stretch parameter *β* < 1) around an average value τ. A plot of the time constant τ as a function of temperature can be fitted to a straight line (Fig. 3e) described by the equation

2 $$\tau = \tau_{0} \exp \left( {E_{\tau } /k_{B} T} \right),$$

where T is the temperature in kelvins and k_B_ is the Boltzmann constant. This equation is typical of thermally activated processes with an activation energy E_τ_. Here, the E_τ_ required to reverse the formation of the defects created during the deep UV light radiation is estimated to be approximately 0.99 eV. This value is close to the activation energy previously obtained for the creation of a V_O_ related double donors during NBIS^25^.

### Location of defects

**Figure 4:**
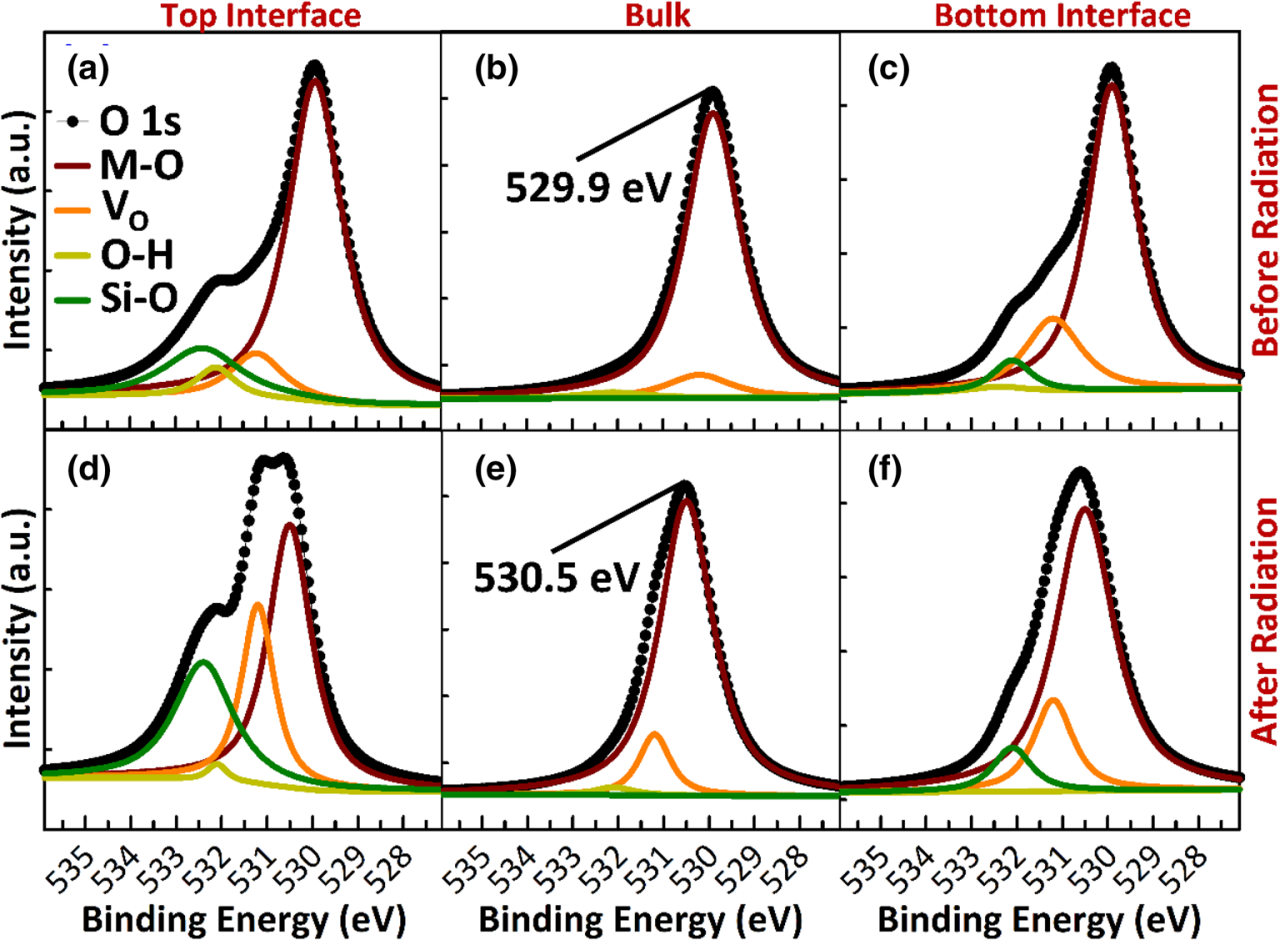
XPS results showing O 1 s spectra of thin-film stacks (glass/SiO_2_/a-IGZO/SiO_2_) before (**a**–**c**) and after (**d–f**) deep UV light exposure at the top surface (**a** and **d**), in the bulk (**b** and **e**), and at the bottom surface (**c** and **f**) of the a-IGZO film. The O 1 s spectrum is deconvoluted into four energy peaks: M–O, V_O_, O–H, and Si–O. Peak O–M, which is centered at 529.9 eV, is attributed to O^2−^ ions binding with In, Ga, and Zn atoms—and thus represents the quantity of the oxygen atoms in a fully oxidized stoichiometric environment. The V_O_ peak at 531.2 eV stems from the deficiently bonded oxygen in the a-IGZO layer containing nonstoichiometric oxide species, such as In_2_O_3−x_, Ga_2_O_3−x_, and ZnO_1−x_, which are associated with V_O_. Peak O–H is related to metal–OH (hydroxyl) bonds and centered at 532.1 eV, while Peak Si–O is related to Si–O bonds and centered at 532.4 eV.

The nature of these defects is investigated by XPS depth profile analysis of thin-film stacks (glass/SiO_2_/a-IGZO/SiO_2_) before and after UV exposure (Fig. 4). The O 1 s spectrum is deconvoluted into four energy peaks: O–M, V_O_, O–H, and Si–O. Peak O–M, which is centered at 529.9 eV, is attributed to O^2−^ ions binding with In, Ga, and Zn atoms—and thus represents the quantity of the oxygen atoms in a fully oxidized stoichiometric environment. Peak O–H is related to metal–OH (hydroxyl) bonds and centered at 532.1, whereas peak Si–O is related to Si–O bonds and centered at 532.4. The V_O_ peak at 531.2 eV stems from the deficiently bonded oxygen in the a-IGZO layer containing nonstoichiometric oxide species, such as In_2_O_3−x_, Ga_2_O_3−x_, and ZnO_1−x_, which are associated with V_O_^26, 27^. Area percentages of each peak before and after radiation are listed in Table 2.

**Table 2:** Area percentages of metal–oxygen (M–O), oxygen vacancy (V_O_), hydroxyl (O–H), and silicon-oxygen (Si–O) peaks from deconvoluted O 1 s spectra.

	M–O (%)		V_O_ (%)		O–H (%)		Si–O (%)	
	Before UV	After UV	Before UV	After UV	Before UV	After UV	Before UV	After UV
Top interface	70	46	10	24	5	2	15	28
Bulk	91	88	7	11	2	1	0	0
Bottom interface	73	70	20	21	1	0	6	8

While the number of Si–O and OH-related defects is negligible in the bulk (Fig. 4b), a significant amount is present at the top (Fig. 4a) and bottom (Fig. 4c) surfaces of the pristine a-IGZO film as expected. The amount of V_O_ is also larger at the surfaces compared to the bulk. Similar situations have been reported before, identifying V_O_ as a surface feature, confined within 0.5 nm of the top surface of the film^16, 28^. While deep UV radiation results in the creation of very few V_O_ defects in the bulk (Fig. 4e) and at the bottom surface (Fig. 4f) of the a-IGZO, a substantial amount is created at the top surface (Fig. 4d), where the area percentage increases from 10 to 24% (Table 2). The area percentage of the Si–O peak also increases from 15 to 28% at the top surface, indicating the diffusion of Si atoms into the a-IGZO layer, possibly from the broken weak Si–O bonds at the top interface. It should be pointed out that a large overlap exists between the energy peaks O–H (532.1 eV) and Si–O (532.4 eV), making it difficult to separate the two. To ensure a reasonable peak ratio, we fixed the full width half-maximum of all components to 1.

The use of optical electron paramagnetic resonance (EPR) experiments has shed light on the nature of V_O_ in ZnO single crystals^29, 30^. V_O_ bind two electrons in their electrically neutral state (V_O_^0^) but can also exist in a singly ionized (V_O_^+^) and doubly ionized (V_O_^2+^) state. The increase in the concentration of V_O_ states at the top surface of the a-IGZO film after light radiation indicates photo-creation of the V_O_. However, this increase is too small to account for ΔV_TH_ > 10 V after light radiation (Fig. 1c). A plausible explanation would be the photoionization of existing and newly created V_O_^0^ to V_O_^2+^ states—a process which donates two electrons to E_C_.

The presence of two electrons in V_O_^0^ triggers the surrounding Zn^2+^ ions to move inwardly, reducing the physical size of the vacancy. Consequently, the binding energy increases as the overlapping between the Zn^2+^ wave functions intensifies, such that ε(+ /0) < ε(2 + /0) < ε(2 + / +), where ε(+ /0) is the transition energy from the V_O_^+^ to V_O_ and similarly for the other two cases^31–33^. V_O_ are thus said to have a negative correlation energy U and the V_O_^+^ is unstable due to the presence of either V_O_^2+^ or V_O_^0^ with a lower formation energy but can be observed under nonequilibrium conditions such as under illumination^34–36^. This means that only the presence of V_O_^0^ or V_O_^2+^ is expected at thermal equilibrium.

The transition from V_O_ to V_O_^2+^ is thus accompanied by lattice relaxation that raises the energy level of V_O_^2+^ after photoionization and leads to metastability of the V_O_^2+^ states. In fact, this lattice relaxation can make energy levels of the V_O_^2+^ states degenerate with E_C_^31, 32^. If that occurs, free electrons occupying lower lying states in the conduction band minimum will need to be activated, thermally or otherwise, to these higher V_O_^2+^ state levels for the reverse reaction, making the V_O_^2+^ a metastable donor defect. Being ionized states that are degenerate with the E_C_, the V_O_^2+^ will not be detected by XPS^28^. However, the negative ΔV_TH_ increase in SS after light radiation is consistent with the photo-creation of shallow states. Moreover, the binding energies of the In3d_5/2_, Ga2p_3/2_, and Zn2p_3/2_ peaks all show a shifting towards higher binding energy, indicating a decrease in the electron density around the metals (Supplementary Fig. S2). This is consistent with ionization.

Hydrogen also plays a role in the instability of the a-IGZO TFTs. In fact, a high concentration of hydrogen (in the order of 10^20^ cm^−3^) has been observed in a-IGZO thin films, without intentional exposure of the films to hydrogen during their deposition^37, 38^. Incorporated hydrogen can fill V_O_ sites in a-IGZO films, forming stable + 1 charge states as donors^39^. If these states are also degenerate with the E_C_, they will be hard to detect by XPS. However, a significant increase in the amount of hydrogen after UV light radiation is detected by ToF–SIMS depth profiling of the SiO_2_/IGZO/SiO_2_ structures used in this study. The increase is mainly confined to the top surface of the a-IGZO layer as can be seen in Supplementary Fig. S3d. Additionally, ToF–SIMS also detected a smaller amount of gallium, zinc, and oxygen at the top interface compared to the bulk (Supplementary Fig. S3), which is consistent with a larger population of defects at the top interface.

Hydrogen impurities generate two types of defects, depending on whether they are bonded to an oxygen (OH) or to a metal (M-H) site. The OH defects have negative formation energy, implying a spontaneous formation whenever hydrogen is present. These defects do not generate states in the gap but act as donors until a high electron concentration is achieved, after which hydrogen starts to bind itself to metal sites, forming acceptors which compensate for the surplus of electrons in the a-IGZO. As the M-H bond requires an electron to form, it thus helps to limit the carrier concentration in a-IGZO, and forms states just above the E_V_^40^. ToF–SIMS results suggest the bonding of hydrogen and indium, given that both significantly increased at the top interface after UV light radiation (Supplementary Fig. S3).

### Origin of the subthreshold hump

The XPS analysis has reviewed that the top surface of the a-IGZO layer is the most affected by light radiation, showing a sizeable increase in the number of V_O_ and Si–O defects. Additionally, calculations for In_2_O_3_ crystals have shown that V_O_ can behave as shallow donors when located at the surface rather than in the bulk^41^. Furthermore, the positive shift in binding energy of the In3d_5/2_, Ga2p_3/2_, and Zn2p_3/2_ peaks is mainly confined to the top half of the a-IGZO film (Supplementary Fig. S2) and a linear plot of the transfer characteristics in Fig. 1c shows two different slopes for negative and positive V_GS_ (Supplementary Fig. S4), which clearly indicates the presence of two logical channels with two different resistances. Taking all this into consideration, the formation of the subthreshold hump in the transfer characteristics after deep UV radiation can thus be explained by the creation of shallow donors at the top surface of the a-IGZO film. This will create a parasitic channel (backchannel) with a conductance that is higher than that of the a-IGZO bulk (frontchannel)^42^.

**Figure 5:**
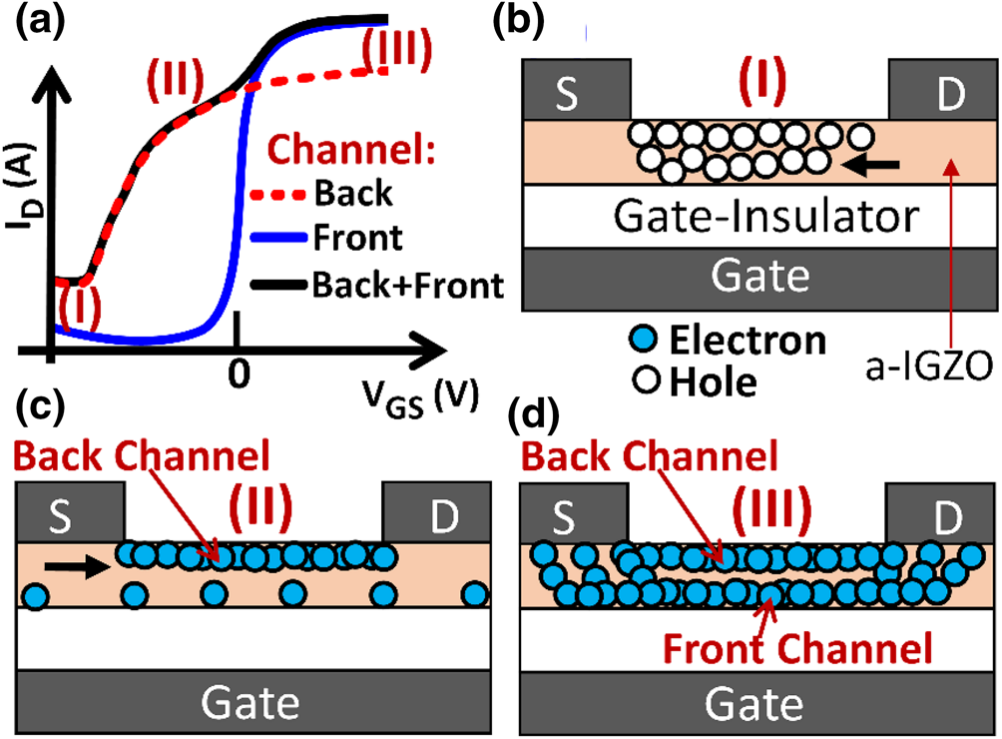
Mechanism for “hump” formation in the transfer characteristics after deep UV light (λ = 172 nm) radiation. (**a**) Schematic transfer characteristics showing the contributions of the back and front channel currents to the total current (Back + Front). (**b**) Off-state current increases due to the flow of holes from the drain (D) to the source (S) when V_GS_ is negative (region I). (**c**) Only the backchannel is conducting when V_GS_ is close to zero volts (region II). (**d**) Both the back and front channels are conducting when V_GS_ is positive (region III).

Figure 5 shows how this conductive backchannel is manifested as a hump in the transfer characteristics of the a-IGZO TFTs post radiation. In Fig. 5a, the transfer characteristics are divided into three regions, labeled I, II, and III. In region I, the a-IGZO layer is depleted of electrons due to the strong negative V_GS_ and only holes remain in the channel. In a-IGZO, it is difficult to induce holes with negative V_GS_, owing to the large concentration of V_O_ located less than 1 eV above E_V_^43^. So, these holes must be the photogenerated holes that have not yet recombined. The holes drift towards the source (S) under the influence of the positive V_DS_, resulting in a small hole current and lowering of the source barrier for electron injection (Fig. 5b)^23, 44^. In region II, electron accumulation begins at the top surface of the a-IGZO, and the backchannel becomes conducting, while the frontchannel is still not (Fig. 5c). In region III, both the front and backchannel are conducting, and I_D_ is the sum of the currents flowing in the two channels (Fig. 5d).

**Figure 6:**
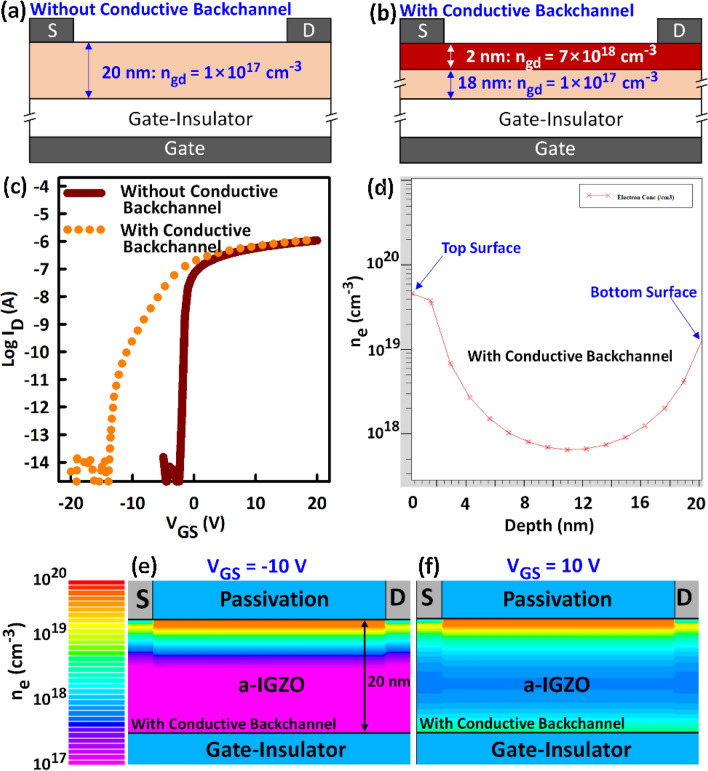
TCAD simulation of the effect of deep UV light radiation on a-IGZO TFTs. (**a** and **b**) Schematics representing the TFT models used to simulate TFT characteristics (**a**) before and (**b**) after deep UV light radiation. A 2 nm thick a-IGZO film with a donor concentration (n_gd_) of 7 × 10^18^ cm^-3^ is used to simulate the photoionization of neutral oxygen vacancies at the top surface of the a-IGZO film. n_gd_ for the bulk is 1 × 10^17^ cm^-3^. (**c**) Simulated transfer characteristics. (**d**) Line profile of the electron concentration (n_e_) from the top to the bottom surface when V_GS_ = 10 V. (**e** and **f**) Images showing the distribution of n_e_ in the a-IGZO with a conductive backchannel when (**e**) V_GS_ =  − 10 V and (**f**) V_GS_ = 10 V. The presence of a conductive top surface induces a negative ΔV_TH_ and the “hump”.

In region III, the current due to the backchannel is lower than that of the bulk (front) (Fig. 5a) because the latter is thicker than the former. TCAD simulation of the TFT transfer characteristics with (Fig. 6a) and without (Fig. 6b) a highly conductive top surface of the a-IGZO film, also yielded the same results (Fig. 6c), verifying the hump formation mechanism. Here, a 2 nm-thick a-IGZO layer with a donor concentration (n_gd_) of 7 × 10^18^ cm^−3^ is used to represent the highly conductive a-IGZO top surface (Fig. 6b). n_gd_ of the bulk is 1 × 10^17^ cm^−3^. Other density of states parameters have the same values as those reported in^45^. A depth profile analysis of the electron concentration (Fig. 6d) indicates that a highly conductive backchannel is responsible for the negative ΔV_TH_. Consistent with the mechanism for the “hump”, only the backchannel is conductive when the V_GS_ is negative (e.g., V_GS_ = − 10 V) as shown in Fig. 6e. When the V_GS_ is positive (e.g., V_GS_ = 10 V), conduction occurs in both the backchannel and frontchannel (Fig. 6f).

It is important to note that the hump starts at the same point (at approximately V_GS_ = 0 V), regardless of the UV light exposure time, although it progressively stretches into the negative V_GS_ direction with increasing exposure time (Supplementary Fig. S4). This is consistent with the increase in the number of photoionized V_O_ at the top surface of the a-IGZO film and the consequent increase in the backchannel current with UV exposure time. In other words, the negative ΔV_TH_ is mostly due to defects generated at the top surface of the a-IGZO film. Application of negative V_GS_ during light illumination pushes the E_F_ towards E_V_ and increases the concentration of holes, favoring the formation of these defects, which is why the effect is larger under NBIS.

It is expected for one to suspect that the results presented herein show defect generation only at the top surface because absorption of light with high photon energy (~ 7.2 eV) is limited to the top surface. However, the penetration depth for λ = 172 in a-IGZO has been reported to be 30 nm for intensity 8 times smaller than the one used herein^46^. In fact, by considering the a-IGZO film thickness (d) of 20 nm and incident light intensity (I_0_) of 400 mW/cm^2^, the transmitted light intensity (I_t_) can be estimated from

3 $$I_{t} = I_{0} e^{ - \alpha d}$$

to be approximately ~ 54 mW/cm^2^, which is too high to eliminate absorption at the bottom surface of the a-IGZO. Here, α is the absorption coefficient, which is assumed to be approximately 10^6^ cm^−1^ for λ = 172 nm based on the extrapolation of previously published results^47^. Therefore, the origin of the light instability in a-IGZO TFTs is indeed due to the ionization of V_O_ states that are intrinsic to the top surface of the a-IGZO films.

### Defect creation mechanism

Thermodynamic calculations have estimated the formation energy of the V_O_^0^ in a-IGZO to be approximately 4 eV^48^, which is too high for the V_O_ to be present in large amounts in native films, consistent with the bulk of the a-IGZO films presented herein (Fig. 4c). However, 4 eV is less than the photon energy of the deep UV light (~ 7.2 eV), making their photo-creation possible. Furthermore, V_O_ can be created from centers with weak or broken bonds in the presence of light, due to the release of energy from electron–hole recombination. Similarly, ε(0/2 +) can be greatly reduced through recombination of the electrons bound to the V_O_^0^ with photogenerated holes, resulting in V_O_^2+^.

The above-mentioned mechanism is similar to the Staebler Wronski effect in a-Si:H^49^, where hydrogen plays a major role in a mechanism known as bond switching^50^. In this case, a weak Si–Si bond is also broken by using the energy released from the recombination of photo-generated electron–hole pairs, and two stable dangling bonds can be formed if a hydrogen atom from a neighboring Si–H bond is exchanged for one of the created dangling bonds. Therefore, atomic exchange after photo-creation of defects in a-IGZO with weak bonds present in the random lattice could play a role in the photo-creation of defects also in a-IGZO. Note that metal vacancies or interstitials are also present in the amorphous network of the IGZO^35^. Additionally, as hydrogen in the excess of ~ 10^20^ cm^-3^ has been detected in a-IGZO^37^, a significant amount of OH and V_O_-related species are apparent at the top surface of the a-IGZO films presented herein.

**Figure 7:**
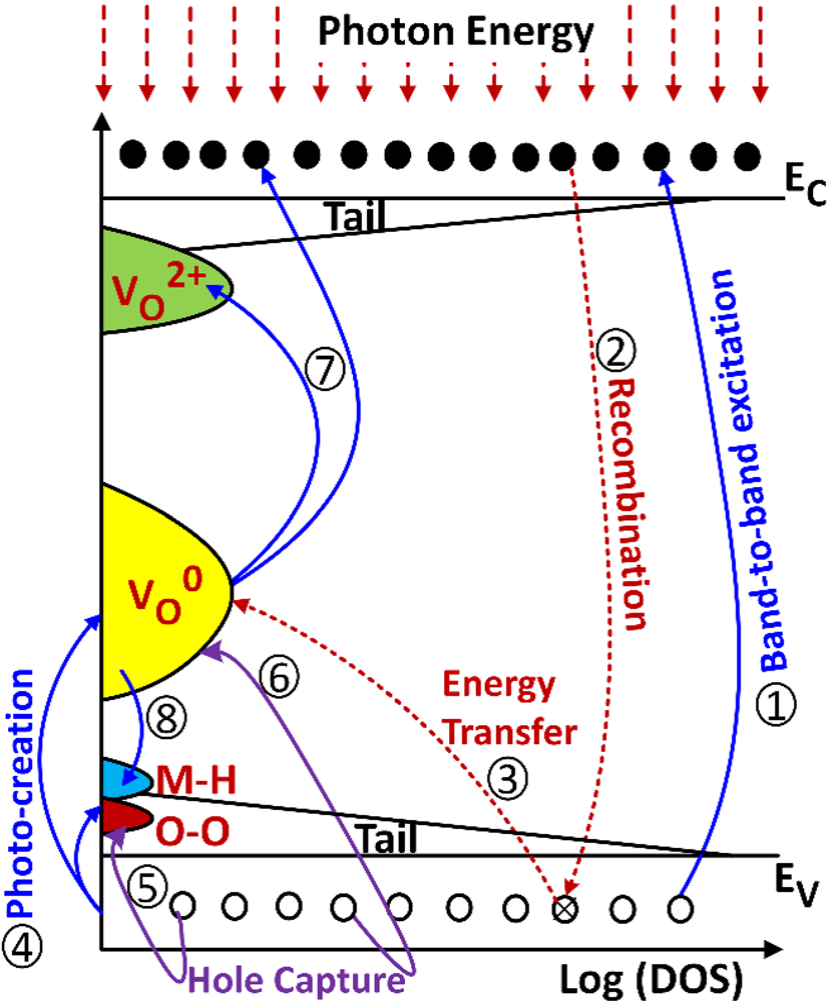
Defect creation under deep UV light illumination. (1) Band-to-band excitation. (2) Electron–hole (e–h) recombination. (3) Breaking of weak bonds to form neutral oxygen vacancies (V_O_^0^) using energy released from e–h recombination and from the heat lost during the descent of electrons from higher to lower states in the conduction band (E_C_). (4) Photo-creation of V_O_^0^ due to the high energy photons. (5) Formation of peroxides (O–O) via hole capture or bonding of two interstitial oxygen atoms. (6) Ionization of V_O_^0^ to V_O_^2+^ via hole capture. (7) Photoionization of V_O_^0^ to V_O_^2+^. Structural relaxation raises the energy level of V_O_^2+^ relative to V_O_^0^. (8) Formation of metal-hydrogen bonds (M–H) via electron capture. This event controls the maximum electron concentration. The numbers do not represent the order in which these events occur.

After photo-creation of V_O_, the oxygen can be accommodated interstitially or through the formation of peroxides, which involve the covalent bonding of two oxygen atoms (O–O). While interstitial oxygens are stoichiometric defects which act as acceptors^34^, peroxides are donors that spontaneously form when a large concentration of holes is available in the E_V_^41^. The mechanism responsible for the deep UV light instability in a-IGZO is thus summarized diagrammatically in Fig. 7.

### Suppression of the light instability

**Figure 8:**
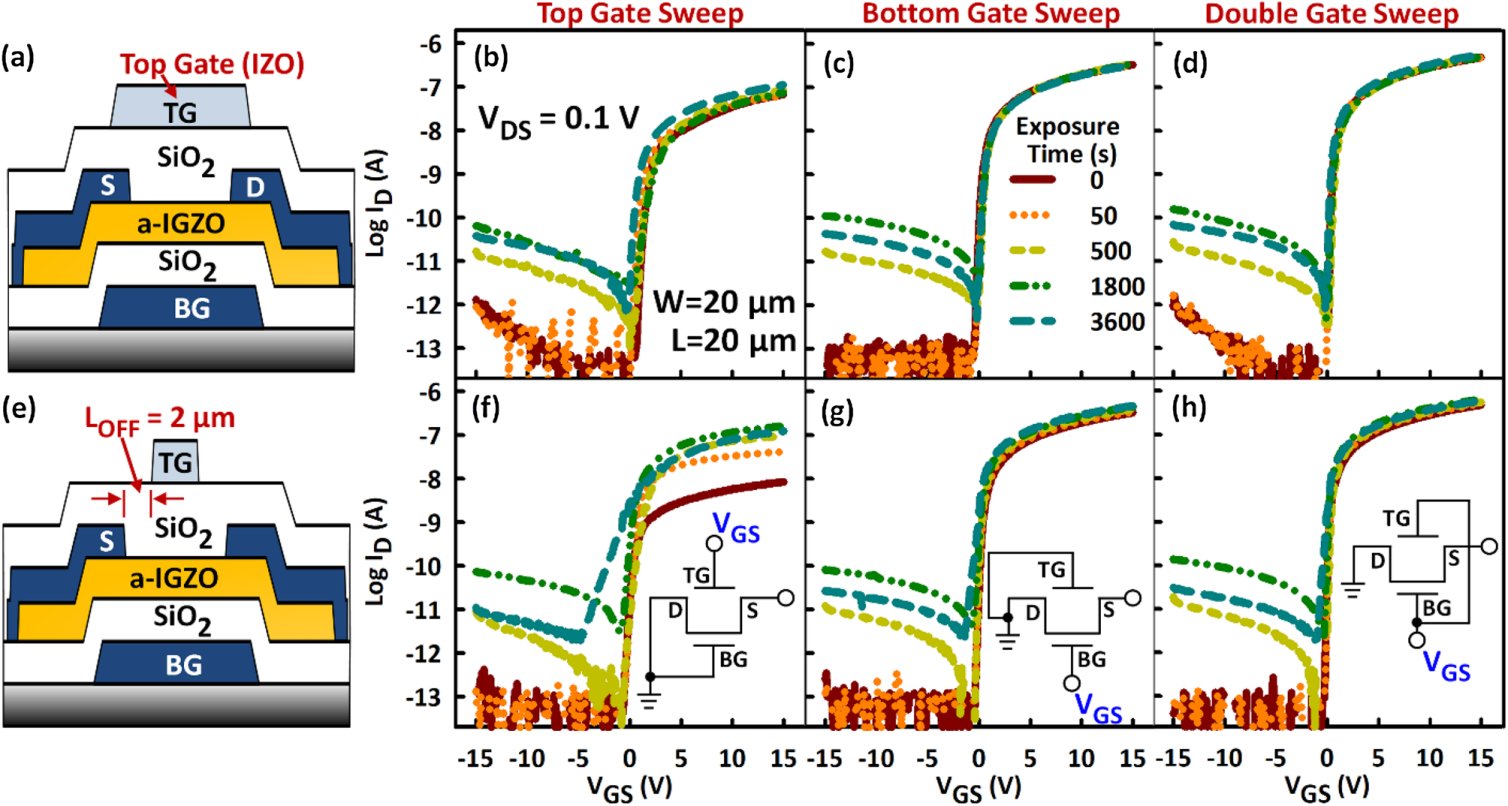
Effect of back bias on deep UV light (λ = 172 nm) instability in a-IGZO TFTs. (**a** and **e**) Double gate TFT structures used for the investigation with a (**a**) full and (**e**) partial back gate (top gate). A transparent top gate (TG) material (IZO) is used to separate the bias from the light-shielding effect. (**c** to **h** and **f** to **h**) Evolution of transfer characteristics with light exposure time under three bias conditions: (**b**) and (**f**) Top gate (TG) sweep, where the TG is swept while grounding the bottom gate as shown by the inset of (**f**). (**c**) and (**g**) BG sweep, where the BG is swept while grounding the TG as shown by the inset of (**g**). (**d**) and (**h**) Double gate (DG) sweep, where TG and BG are shorted as shown by the inset of (**h**). Grounding or applying a positive back bias after radiation suppresses light instability in a-IGZO TFTs.

Having identified the backchannel as the source of most of the light instability, owing to the defects that occupy the top surface of the a-IGZO, the effect of applying a back bias to reduce the instability is investigated. Double gate TFT structures with transparent top gates that fully (Fig. 8a) or partially (Fig. 8e) cover the channel region are used in the investigation. A transparent material (IZO) is intentionally used for the top gates to isolate the effect of the electrical back bias from that of light-shielding. Three bias conditions are investigated: (1) Top gate (TG) sweep, where the TG is swept from − 15 to 15 V, while grounding the bottom gate (BG) (see inset of Fig. 8f). (2) BG sweep, where the BG is swept from − 15 to 15 V, while grounding the TG (see inset of Fig. 8g). (3) Double gate (DG) sweep, where the TG and BG are shorted and swept from − 15 to 15 V (see inset of Fig. 8h).

Extracted TFT parameters can be found in Supplementary Fig. S5. The DG TFT results are interesting and support the light instability mechanism described above. First, the existence of a full TG bias suppresses the negative ΔV_TH_, for all bias conditions (see Fig. 8b–d and Supplementary Fig. S5a). However, the off-state leakage current (I_OFF_) significantly increases after UV light radiation (Fig. 8 and Supplementary Fig. S5b). This indicates that I_OFF_ is the result of photogenerated holes drifting from the D to the S. While a grounded or negatively biased TG suppresses electrons from the conductive backchannel, which is the reason for the negligible ΔV_TH_, it promotes hole induction, consistent with the high I_OFF_. I_OFF_ is also high during the DG sweep (grounded TG) because the hole layer is simply shifted towards the frontchannel, which is negatively biased. Grounding or negatively biasing the TG does not make a big difference with regards to the size of the off-state current because hole induction by V_GS_ is difficult in a-IGZO, owing to the large concentration of V_O_ located less than 1 eV above E_V_^43^. The holes responsible are the photogenerated holes that are yet to recombine.

Second, negative ΔV_TH_ and increase in SS occur after UV light radiation for 3600 s when a partial TG is implemented (Fig. 8f and Supplementary Fig. S5c). However, the negative ΔV_TH_ is not as large as that of a single gate TFT (Fig. 1c), indicating partial suppression of the conductive backchannel electrons by the partial TG. Additionally, the SS increases without the formation of a hump during the TG sweep (Fig. 8f) because there is only one channel (the backchannel) involved, supporting the mechanism for the “hump”. However, suppression of the effect of the conductive backchannel electrons can be seen during the BG (Fig. 8g) or DG (Fig. 8h) sweep with a partial TG but evidence of it just starting to appear is apparent. The increase in the on-state current (I_ON_) after UV light radiation during the TG sweep (Fig. 8f) is consistent with a photoinduced increase in the conductance of the offset regions. Note that the increase in I_ON_ after UV light radiation is almost negligible when a full TG is implemented (Supplementary Fig. S5b). Consequently, the change in field-effect mobility after UV light radiation is exceedingly small when a full TG is used (Supplementary Fig. S5d).

**Figure 9:**
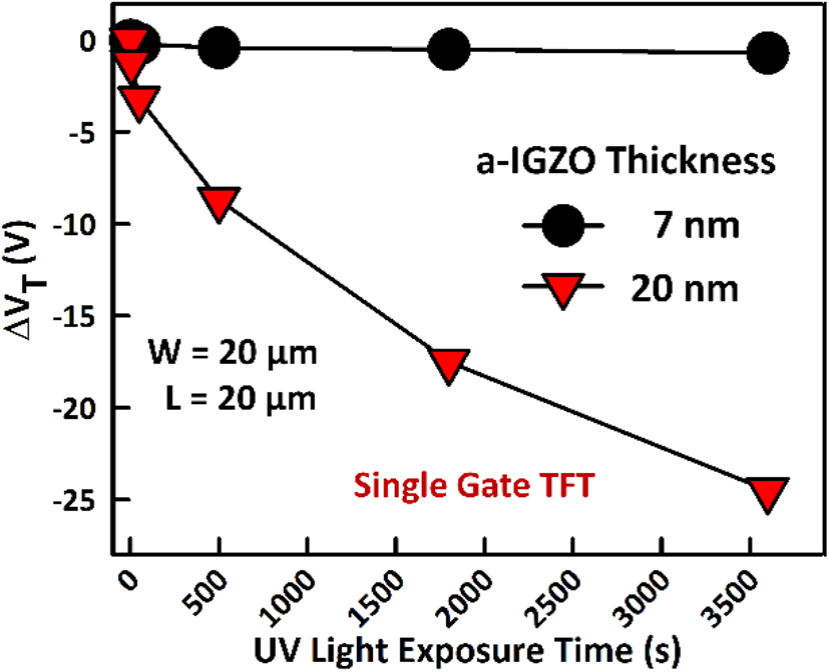
Threshold voltage shift (ΔV_TH_) in inverted staggered (single gate) a-IGZO TFTs as a function of deep UV light (λ = 172 nm) exposure time. Reducing the thickness of the a-IGZO from 20 to 7 nm almost completely suppresses the deep UV light instability. a-IGZO film thickness reduction enhances the bottom gate control over the back (top) channel and also decreases the total number of defects.

These results provide further evidence showing that defects occupying the top surface of the a-IGZO are the source of the light instability in a-IGZO TFTs and that their effect can be suppressed by applying a back bias. This is especially important in applications such as transparent displays where the use of light shields is not possible. Although back biasing does not prevent the off-state currents from increasing, this is not expected to be a problem as they do not exceed 100 pA and they quickly recover at room temperature when the holes recombine. An alternative to applying a back bias, for instance in single gate inverted staggered TFTs (Fig. 1a), would be to reduce the thickness of the a-IGZO film. By doing this, the bottom (front) gate will not only have control of the backchannel, but the total number of defects is also reduced^9^. As shown in Fig. 9, using an a-IGZO film thickness of 7 nm results in negligible ΔV_TH_.

Fabrication process optimization to ensure a clean back interface is thus another way to minimize the light instability. The use of etch-stopper layers^51^, damage-free source/drain metal etchants^19^, high quality encapsulation layers^16^, high temperature or long-time annealing^12^, high pressure or water vapor-assisted annealing^17, 18^ and treatments^52, 53^, are some of the optimization techniques that can be implemented.

## Conclusion

By radiating a-IGZO TFTs with deep UV light (λ = 175 nm), we have shown that the origin of light instability in a-IGZO TFTs is V_O_ and hydrogen-related defects that are located at the top surface of the a-IGZO film. Under light illumination, metastable donor states of these defects are created, making the backchannel conductive. This conductive backchannel results in rigid negative ΔV_TH_ that is accompanied by an increase in SS and a ‘hump’ in the subthreshold region of the transfer characteristics. Most importantly, we have also shown that this instability to light illumination can be suppressed by applying a back bias or decreasing the thickness of the a-IGZO film in applications, such as transparent displays in Heads Up Displays (HUDs) or smart glasses, where the use of light shields is not permissible. Fabrication process techniques that ensure a clean top surface of the a-IGZO film, such as plasma treatments, water vapor-assisted annealing, and high-quality encapsulation layers are also necessary to minimize the light instability.

## Methods

### Device fabrication

We fabricated the a-IGZO TFTs with the inverted staggered structure (Fig. 1a) using the backchannel etch (BCE) process described in detail elsewhere^19^. In this process, molybdenum (Mo) layers deposited by sputtering at 200 °C are used for the gate (60 nm) and source/drain (150 nm) electrodes. Silicon-dioxide (SiO_2_) layers deposited through plasma-enhanced chemical vapor deposition at 360 °C and 200 °C are used for the gate-insulator (200 nm) and the passivation layer (200 nm), respectively. The 20 nm-thick a-IGZO layer is deposited at 200 °C by reactive sputtering using a polycrystalline IGZO target (In_2_O_3_:Ga_2_O_3_:ZnO = 1:1:1 mol%). The gate-insulator (G.I.) and the a-IGZO layer are deposited consecutively in a cluster deposition tool, without breaking vacuum, to achieve a clean G.I./a-IGZO interface. A hydrogen peroxide-based etchant (pH =  ~ 5) is used to pattern the source/drain electrodes to minimize backchannel acid corrosion^19^, and to ensure a reproducible unstressed state, the devices are annealed at 250 °C in vacuum for 2 h before measuring.

### Characterization

We used the Agilent 4156C precision semiconductor parameter analyzer and the Agilent E4980A Precision LCR meter to measure the current–voltage (I–V) and capacitance–voltage (C–V) characteristics, respectively. For the C–V measurement, we superimposed the DC gate-voltage (V_GS_) on a small AC signal (0.1 V) of frequency 1 kHz, while keeping the source and drain shorted. We derived the field-effect mobility (µ_FE_) in the linear regime (with drain voltage (V_DS_) = 0.1 V) from the transconductance (gm = ∂I_D_/∂V_GS_) by using µ_FE_ = (gm*L)/(W*C_OX_*V_DS_). Here, I_D_, C_OX_, L, and W are the drain terminal current, the G.I. capacitance per unit area, the channel length, and the channel width, respectively. We took the SS as the minimum value of (∂log(I_D_)/∂V_GS_)^−1^ and the threshold voltage (V_TH_) as the V_GS_ corresponding to I_D_ of 1 nA. We carried out the light illumination experiments at room temperature with the TFT electrodes in the floating state. To further characterize the effects of deep UV light radiation, we extracted flat band parameters by a combined analysis of the TFT transfer (I_D_–V_GS_) and C–V characteristics according to methods previously described in^20^. We characterized thin film stacks of SiO_2_ (100 nm)/a-IGZO (20 nm)/SiO_2_ (100 nm) by using Time-of-Flight Secondary Ion Mass Spectrometry (ToF–SIMS) and X-ray photoelectron spectroscopy (XPS) depth profiles. For ToF–SIMS, an ION-TOF (Münster, Germany) instrument (TOF–SIMS V) equipped with a Bi1 + (30 keV, 1 pA) and Cs + (3 keV, 30 nA) gun is used and raster areas for sputter and analysis are 200 μm × 200 μm and 50 μm × 50 μm, respectively.

## Supplementary Information

Supplementary Information.

## Data Availability

The authors confirm that all the data supporting the findings of this study are available within the article [and/or] its supplementary materials.
