# Supplementary material for: Origin of light instability in amorphous IGZO thin-film transistors and its suppression
Source: Sci Rep. 2021 Jul 16;11:14618. doi: 10.1038/s41598-021-94078-8 (PMC8285384; doi:10.1038/s41598-021-94078-8)
Supplement: Supplementary file 1 — Supplementary Information. [file 41598_2021_94078_MOESM1_ESM.docx]

**Supplementary Information**

Origin of light instability in amorphous IGZO thin-film transistors and its suppression

Mallory Mativenga^1,*^, Farjana Haque^1^, Mohammad Masum Billah^1^, and Jae Gwang Um^2^

^1^ Department of Information Display, Kyung Hee University, Seoul 02447 South Korea
^2^LG Electronics, Seoul 17709, South Korea ^*^ Corresponding author: Mallory Mativenga (e-mail: [mallory@khu.ac.kr](mailto:mallory@khu.ac.kr)).


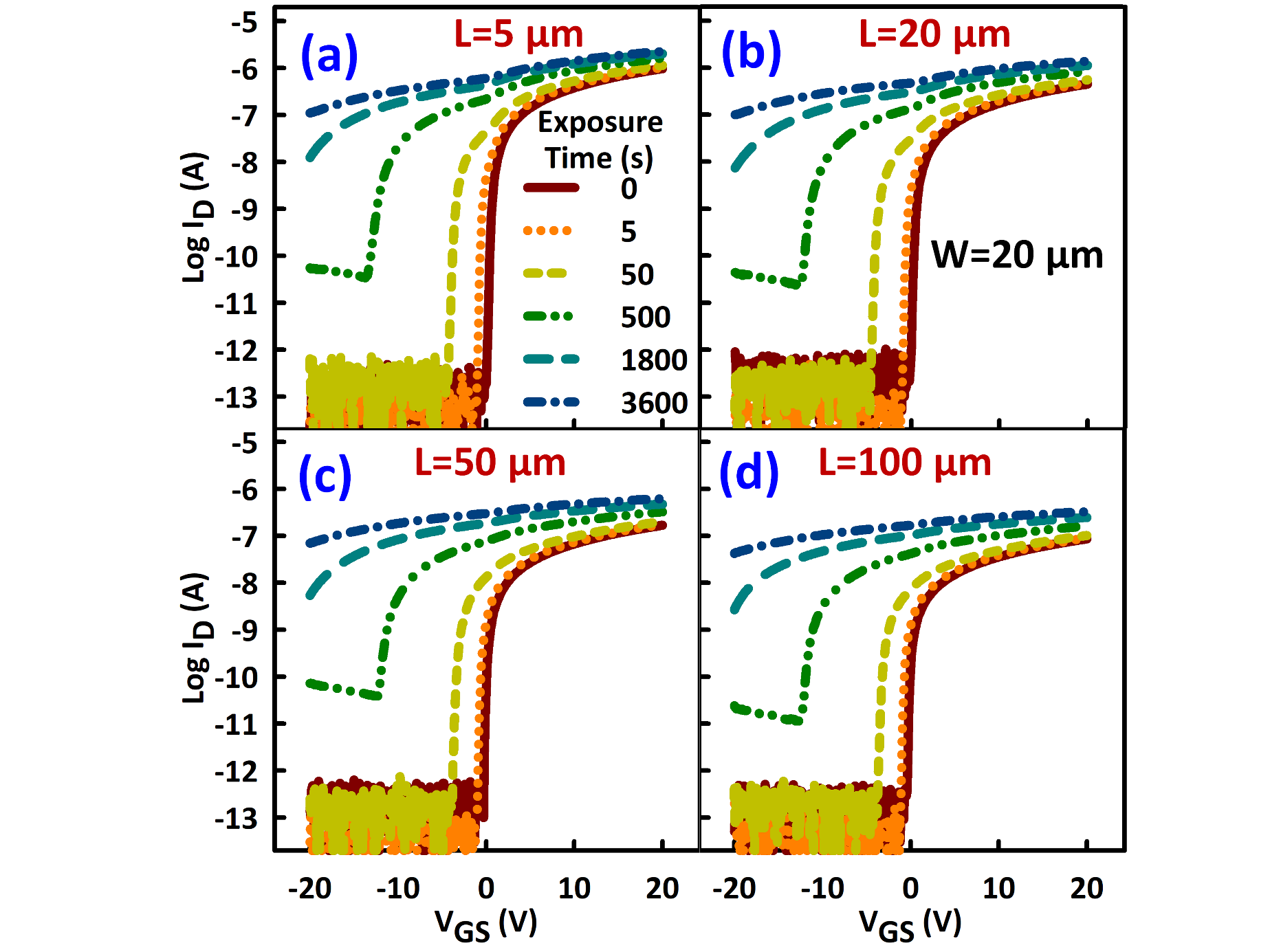


Figure S1.  Transfer characteristics as a function of exposure time to deep UV light (λ=172 nm) of TFTs with channel width (W) of 20 μm and channel length (L) of (a) 5 μm, (b) 20 μm, (c) 50 μm, and (d) 100 μm. The effect of deep UV light radiation is reproducible over many samples and independent of channel dimensions.


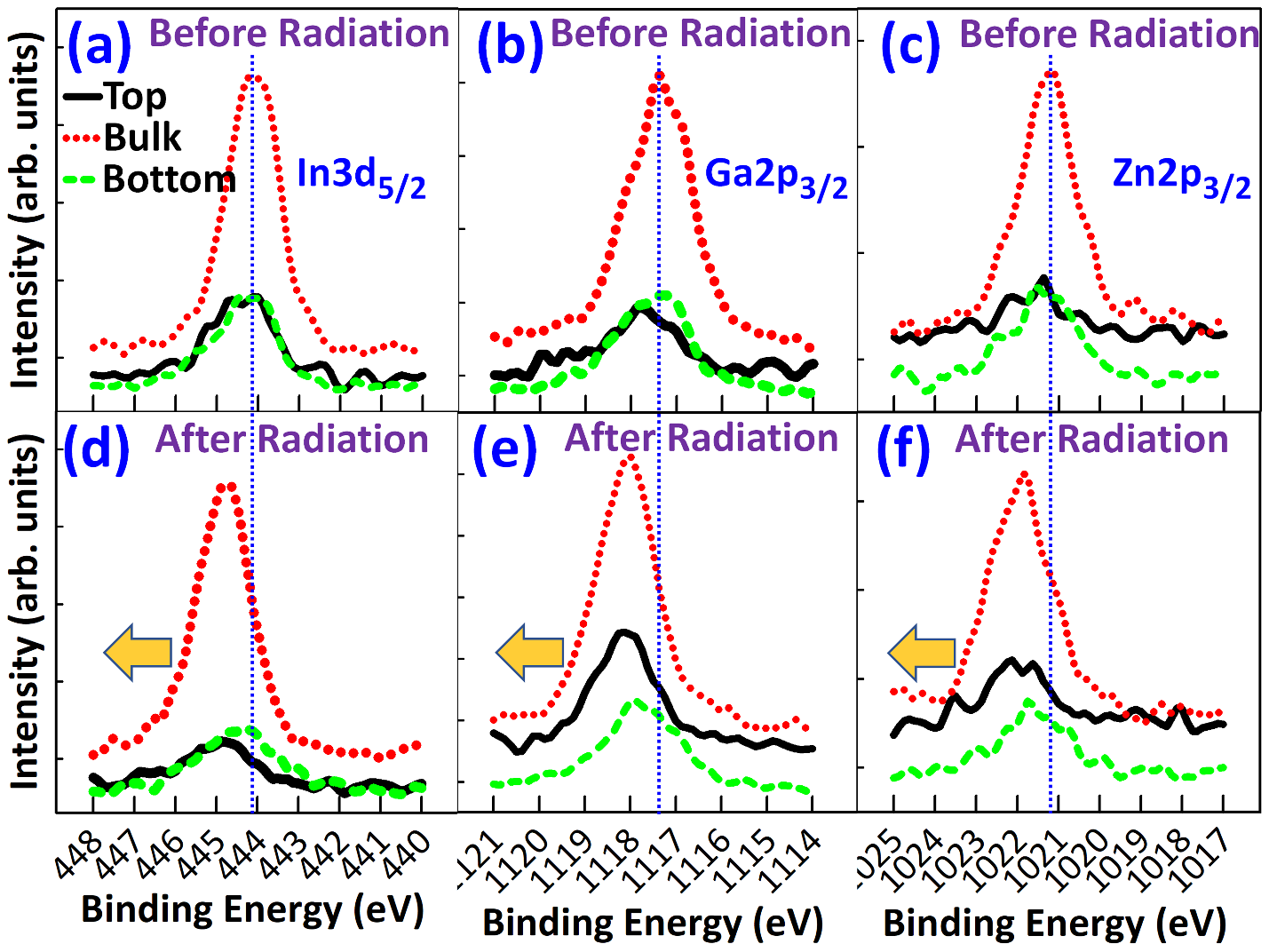


Figure S2.  XPS results showing (a and d) In3d_5/2_, (b and e) Ga2p_3/2_, and (c and f) Zn2p_3/2_ spectra of thin-film stacks (glass/SiO_2_/a-IGZO/SiO_2_) before and after deep UV light exposure.


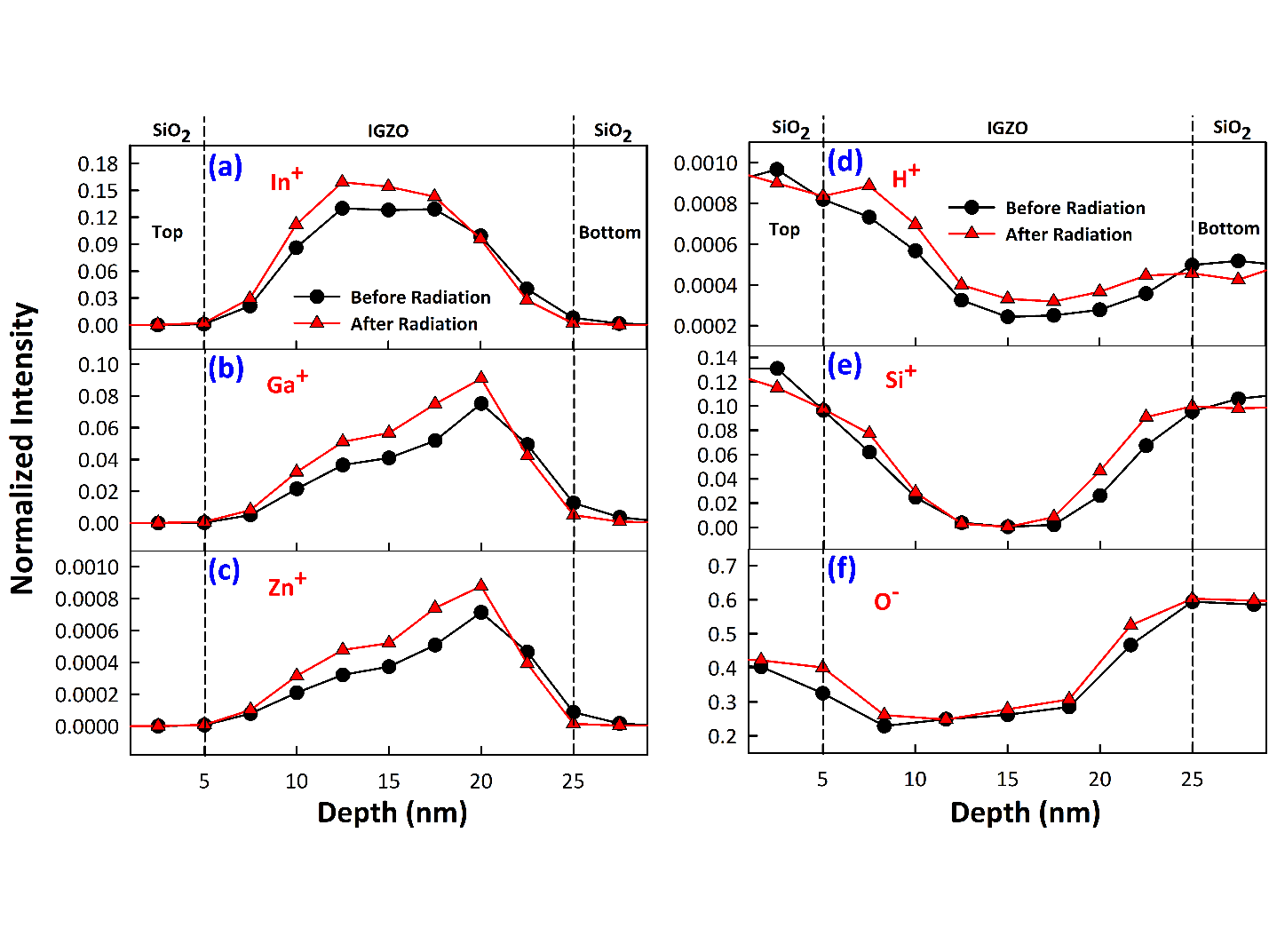


**Figure S3.**ToF-SIMS depth profiles of (a) In^+^, (b) Ga^+^, (c) Zn^+^, (d) H^+^, (e) Si^+^, (f) O^-^ in a thin film layer structures of SiO_2_/a-IGZO/SiO_2_ on glass substrate. The normalized intensity is achieved by dividing the intensity of each element by the total counts at that depth. An ION-TOF (Münster, Germany) instrument (TOF-SIMS V) equipped with a Bi1+ (30 keV, 1 pA) and Cs+ (3 keV, 30 nA) gun is used and raster areas for sputter and analysis are 200 μm × 200 μm and 50 μm × 50 μm, respectively.


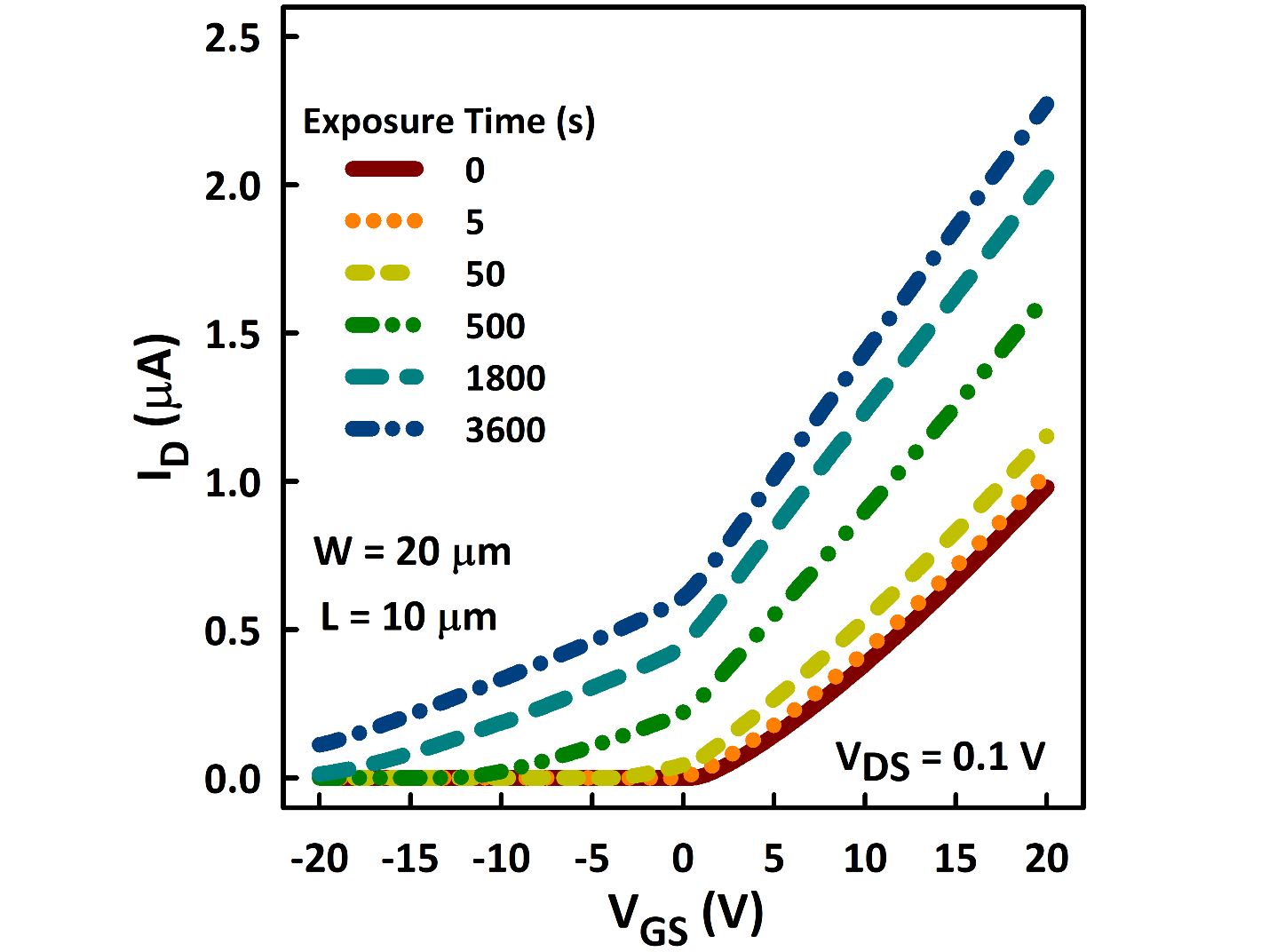


Figure S4. Linear plot of the transfer characteristics in Fig. 1c. The two slopes separating at V_GS_ = 0 V clearly indicate the presence of two logical channels with two different resistances.


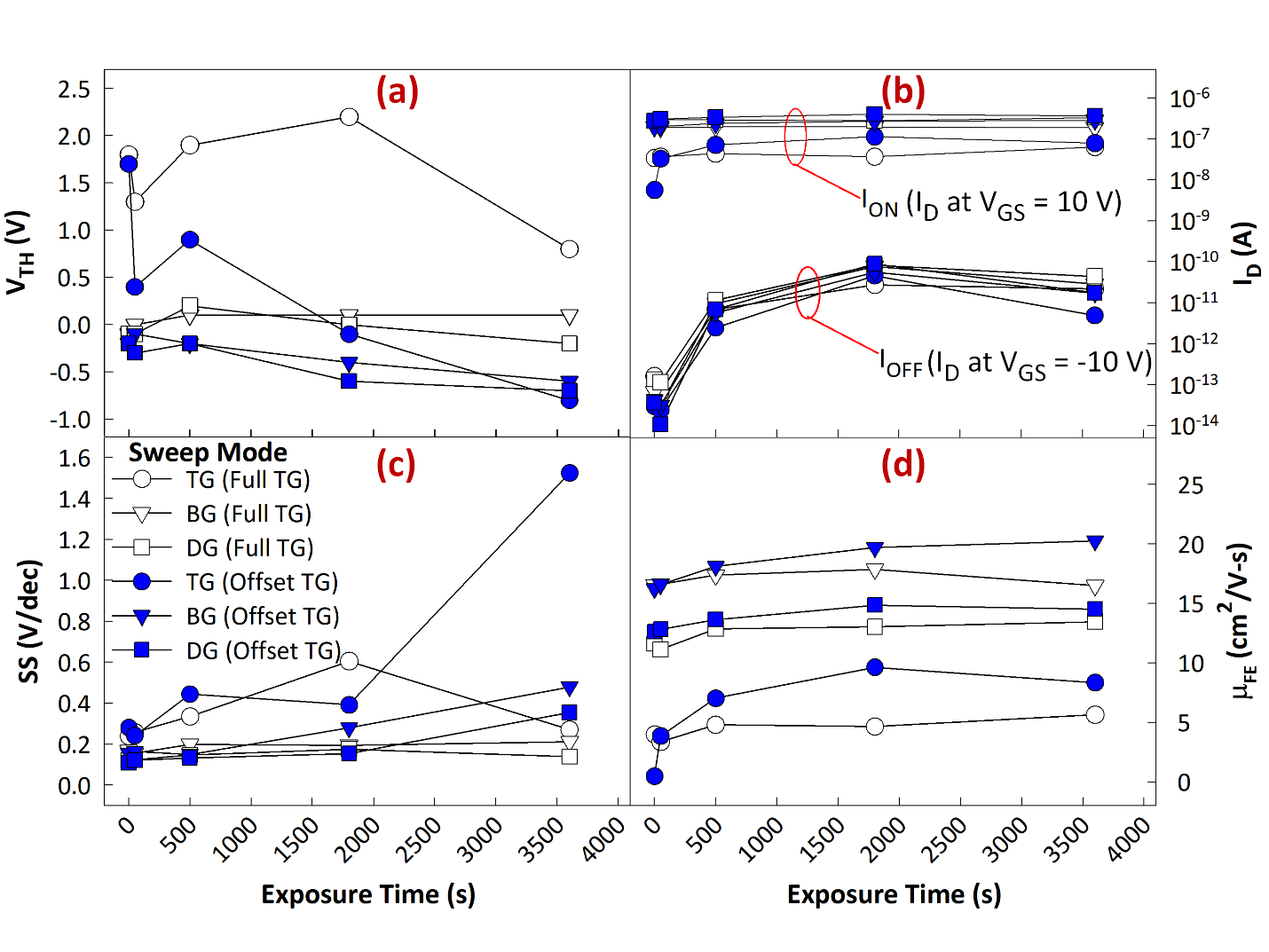


Figure S5. Extracted parameters of double gate (DG) TFTs with full and partial (offset) top gates (TGs) as functions of UV light exposure time. In TG sweep mode, the TG is swept while grounding the bottom gate (BG). In BG sweep mode, the BG is swept while grounding the TG. In DG sweep mode, the TG and BG are shorted and swept simultaneously. (a) Threshold voltage (V_TH_), taken as the V_GS_ corresponding to I_D_ of 1 nA. (b) On-state current (I_ON_) and off-state current (I_OFF_). (c) Subthreshold voltage swing (SS). (d) Field-effect mobility (μ_FE_).
